# Robust phase retrieval for high resolution edge illumination x-ray phase-contrast computed tomography in non-ideal environments

**DOI:** 10.1038/srep31197

**Published:** 2016-08-09

**Authors:** Anna Zamir, Marco Endrizzi, Charlotte K. Hagen, Fabio A. Vittoria, Luca Urbani, Paolo De Coppi, Alessandro Olivo

**Affiliations:** 1 University College London, Department of Medical Physics and Biomedical Engineering, London, WC1E 6BT, United Kingdom; 2 University College London, Institute of Child Health and Great Ormond Street Hospital, London, WC1N 1EH, United Kingdom

## Abstract

Edge illumination x-ray phase contrast tomography is a recently developed imaging technique which enables three-dimensional visualisation of low-absorbing materials. Dedicated phase retrieval algorithms can provide separate computed tomography (CT) maps of sample absorption, refraction and scattering properties. In this paper we propose a novel “modified local retrieval” method which is capable of accurately retrieving sample properties in a range of realistic, non-ideal imaging environments. These include system misalignment, defects in the used optical elements and system geometry variations over time due to vibrations or temperature fluctuations. System instabilities were analysed, modelled and incorporated into a simulation study. As a result, an additional modification was introduced to the retrieval procedure to account for changes in the imaging system over time, as well as local variations over the field of view. The performance of the proposed method was evaluated in comparison to a previously used “global retrieval” method by applying both approaches to experimental CT data of a rat’s heart acquired in a non-ideal environment. The use of the proposed method resulted in the removal of major artefacts, leading to a significant improvement in image quality. This method will therefore enable acquiring high-resolution, reliable CT data of large samples in realistic settings.

In the past decade, x-ray phase contrast imaging (XPCi) has been the focus of extensive research, owing to its typical increase in image contrast, especially for samples where conventional, absorption-based radiography has shortcomings, such as low-attenuating materials^1^. The increase in image contrast in XPCi originates from the sensitivity to phase shifts introduced by the sample as the x-ray beam traverses it. These effects are driven by the sample’s complex refractive index, described by:


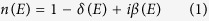


where *E* is the photon energy, and δ and β are linked to phase (refraction) and attenuation effects, respectively. Amongst the different XPCi modalities^2^,^3^,^4^,^5^,^6^,^7^,^8^, Edge Illumination (EI) is a non-interferometric method, well-suited for use in both synchrotrons and laboratories^9^,^10^,^11^. Through dedicated algorithms (termed “phase retrieval”), the method enables the quantitative retrieval of absorption, refraction and ultra-small-angle scattering in the sample^12^,^13^,^14^, and can be implemented as a computed tomography (CT) modality^15^,^16^. The working principle of EI is demonstrated in Fig. 1(a). The incoming x-ray beam is separated into beamlets by a mask positioned immediately before the sample (“sample mask”). The sample mask period is sufficiently large to ensure that the beamlets are physically separated and do not interfere. A second mask (“detector mask”) is placed immediately before the detector, creating insensitive regions between detector pixels. By shifting the sample mask with respect to the detector mask, x-ray refraction induced by the sample is translated into detectable intensity variations. Projection images in EI are of differential nature since the refraction angle is directly proportional to the gradient of the phase shift Φ(*x, y*), according to 
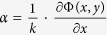
, where *k* = 2*π*/λ and λ is the x-ray wavelength^12^. The phase shift is linked to sample properties and is expressed as^15^: 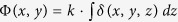. In phase CT however, the reconstructed quantity is δ, and so an integration step is necessary.

It has been shown that in an EI setup, to first approximation the intrinsic spatial resolution in the x-direction is given by the aperture size of the sample mask^17^. However, a high spatial sampling rate is required to fully exploit this high resolution. As can be seen in Fig. 1(a), parts of the sample are not illuminated by the x-ray beamlets, due to the absorbing parts of the sample mask, implying that under-sampling occurs. To overcome this problem, and, thus, to optimally exploit the intrinsic spatial resolution of the EI system, a process called “dithering” can be used, where multiple projections are acquired as the sample is translated along the x-axis by sub-pixel steps. These projections are later recombined to create a higher resolution, “dithered” projection. The increase in resolution however comes at a cost of time and dose, as the total scan time is increased by a factor equal to the number of dithering steps. This consideration becomes especially relevant when CT scans are required, and projections are acquired over at least 180 degrees.

Recently, many activities within our group were targeted at the development of fast and low-dose scanning schemes, often by trading-off spatial resolution^18^,^19^. However, for certain applications (e.g. material science, *ex-vivo* medical research) the requirement of a high spatial resolution may be more important than dose/exposure time considerations, inevitably resulting in long scans. In this paper we present a robust phase retrieval method, developed to provide a solution for long, high resolution EI CT scans in non-ideal environments. The algorithm is based on a “local” retrieval method developed by Endrizzi *et al*.^14^ which has been shown to allow for a significant degree of system misalignment. This new modified “local retrieval” algorithm has been developed specifically for CT scans, which are more sensitive to local misalignments due to the integration step necessary for the retrieval of *δ*. Furthermore, it removes the need to acquire “flat-field” images (i.e. images without the sample), leading to a substantial reduction of scan time compared to the previous method. Importantly, this adaptation incorporates corrections of time-varying system components, whether these are due to vibrations or temperature fluctuations, affecting the source and/or the masks. This is particularly relevant since EI CT is suited for use with commercial x-ray tubes, and so its ability to provide high precision, quantitatively reliable data in environments affected by such instabilities is crucial to its future translation into realistic sites such as hospitals and factories. Possible sources of instability were analysed, modelled, and incorporated into an existing simulation of the EI setup^20^. Experimental results on a complex biological sample are presented, demonstrating a very significant improvement in image quality thanks to the proposed retrieval method.

## Theory

**Figure 1:**
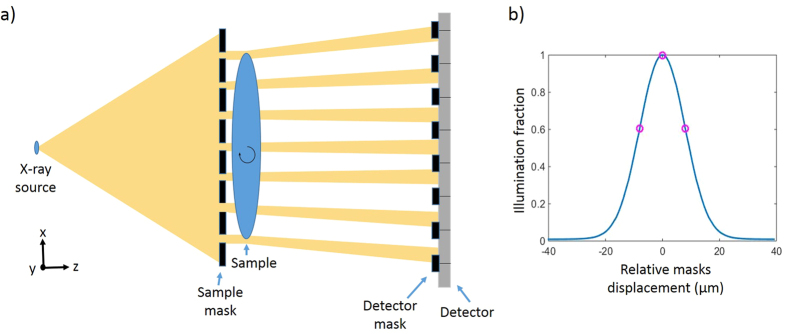
(**a**) A top view of an EI setup with an extended x-ray source. For CT, the axis of rotation is aligned with the y direction, while the sample is moved by sub-pixel steps along x for dithering. (**b**) A typical illumination curve showing intensity variation as a function of masks displacement over one period. The circles represent regular choices of mask positions for imaging.

Current EI phase retrieval methods allow the extraction of three different sample properties; absorption, refraction and scattering. The intensity recorded with the EI setup by a single detector pixel can be described by:


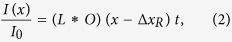


where *I*_*0*_ is the intensity transmitted through the sample mask aperture, *L* is the illumination curve describing the intensity change as a function of relative masks positioning x (see Fig. 1(b)), *O* is the sample’s scattering distribution, Δ*x*_*R*_ is the beam shift due to sample refraction, *t* is the fraction of transmitted intensity through the sample, and * is the convolution operator^12^.

Both *L* and *O* can be represented as a sum of Gaussian functions:


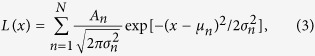



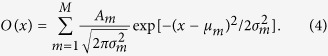


Thus equation (2) can be rewritten as:


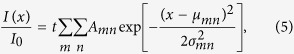


where 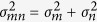, 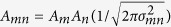 and 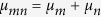.

Equation (5) can be analytically inverted to obtain solutions for absorption, refraction and scattering, in the case where three images are acquired in positions 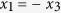, 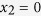 with respect to the illumination curve (“global retrieval”)^13^. When applied to experimental data, it therefore assumes the same positions on the illumination curve for every pixel over the entire field of view, not taking into account any local variations due to misalignment of optical elements or masks imperfections. These variations are mostly compensated for by normalising the raw data by flat-field images.

As has been shown by Endrizzi *et al*.^14^, equation (5) can also be used to retrieve sample absorption, refraction and scattering, without making any assumptions on system alignment. This is done by applying it on a pixel-by-pixel basis, where equation (5) is used as a model function for a non-linear curve-fitting, solved by the least-squares method. This method (local retrieval) allows corrections for system misalignment and mask defects.

While the algorithm described here is based on the local retrieval method, a further extension of the retrieval equation was required to account for system instabilities, and in particular for a lateral shift of the illumination curve. This further development involved the use of information from background regions in the images, to estimate the new parameters of the illumination curve, therefore adding a degree of freedom to the fitting process. The correction term 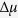, corresponding to the translational shift of the illumination curve, was incorporated into equation (5) in the following way:


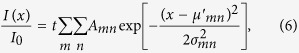


where 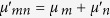, 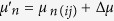, and i and j correspond to individual pixel coordinates.

The illumination curve’s mean position can be calculated for each pixel as *μ*_*n*(*ij*)_ from an initial illumination curve scan. To find the translational shift of the illumination curve over time, let us define a background area in the field of view. This background region could be any part of the field of view which is not covered by the sample throughout its rotation. However, since information from this region is used as correction terms for sample data, it is beneficial that the background region is chosen as close as possible to the sample, whether next to it or above. For each pixel in the background region, the new position of the illumination curve’s mean is estimated as *μ*_*n*_*im*(*ij*)_ by means of a least-squares curve fitting using values from the three images acquired at each dithering step. The shift Δ*μ* is then determined according to:


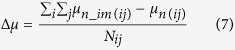


where *N*_*ij*_ is the total number of pixels in the defined background region. The shift Δ*μ* is then applied in the phase retrieval to all pixels in the same dithering step image. Notably, in contrast to the global retrieval, here no flat-field images are needed for processing, as all the required pixel-wise information is drawn from the illumination curve scan.

## Methods

### EI Experimental Setup

Figure 1(a) depicts the EI experimental setup used in the laboratory. The sample and detector masks were placed at 1.6 m and 1.96 m downstream the source, respectively. Both masks were fabricated by electroplating gold strips onto a graphite substrate (Creatv Microtech Inc., Potomac, MD, USA). The sample mask period was 79 μm with an aperture size of 10 μm, while the detector mask period and aperture size were 98 and 17 μm, respectively. The sample was placed immediately after the sample mask, such that the distance between the sample mask and the axis of rotation was approximately 5 cm. The detector mask to detector distance was approximately 4 cm. The source used was a Rigaku MicroMax 007 HF rotating anode (molybdenum) x-ray tube (Rigaku Corporation, Japan) with a focal spot of approximately 70 μm. The detector was a CMOS image sensor C9732DK-11 (Hamamatsu, Japan) with a pixel size of 50 × 50 μm^2^. However, the effective pixel size in the x-direction was 100 μm due to the line-skipping design of the detector mask, where every second detector pixel column is completely covered by the detector mask^21^ and was therefore discarded during data processing.

### Simulation Study

To analyse and understand the effect of system misalignment on the retrieved phase slices, a simulation study was performed. The simulation code is wave-optics based, and is designed to replicate the experimental EI setup used in the laboratory^20^. Input parameters for relative distances and mask characteristics were chosen to match those of the experimental setup. The simulated sample was a cylinder (1 cm diameter) with refractive index values similar to those of the experimental data shown below (in “Results”): δ = 1.7 × 10^−7^ and β = 2.7 × 10^−10^. A photon energy of 17.5 keV was assumed, corresponding to the k-alpha line of the molybdenum target used in the laboratory source. To test the performance of the proposed retrieval method, system misalignment was modelled and incorporated into the simulation. This was done by extracting the illumination curve parameters of each pixel in a detector row from an experimental measurement of the illumination curve. The position of the mean of the illumination curve for each pixel was used to model pixel-wise misalignment in the simulation, by adding it as an offset to the centre position of each aperture in the sample mask. This is equivalent to each pixel featuring the same illumination curve, however centred on a different position. To perform global retrieval, flat-field signals were generated as well, however with a slightly different misalignment, as is the case in an experimental environment. The flats’ misalignment values were taken from the same detector row used for the sample signals, however from a different illumination curve scan. Sinograms were generated at three positions on the illumination curve (at relative masks displacement *x* = −8, 0, +8 μm, see circles in Fig. 1(b), before adding the misalignment offset). To test the global retrieval, sample sinograms were first normalised by the flat sinograms, as would happen experimentally. The normalised sinograms were then processed with the global retrieval algorithm, using the same illumination curve for the entire field of view. To test the modified local retrieval, sample sinograms (no flat normalisation) were processed with the proposed algorithm, using a differently centred illumination curve for each pixel, according to the input misalignment. Sinograms of the sample’s differential phase, absorption and scattering properties were produced from each of the retrieval methods. For CT reconstruction of the phase maps, filtered-back-projection (FBP) was used with the Hilbert filter^22^,^23^.

### Illumination Curve Stability

**Figure 2:**
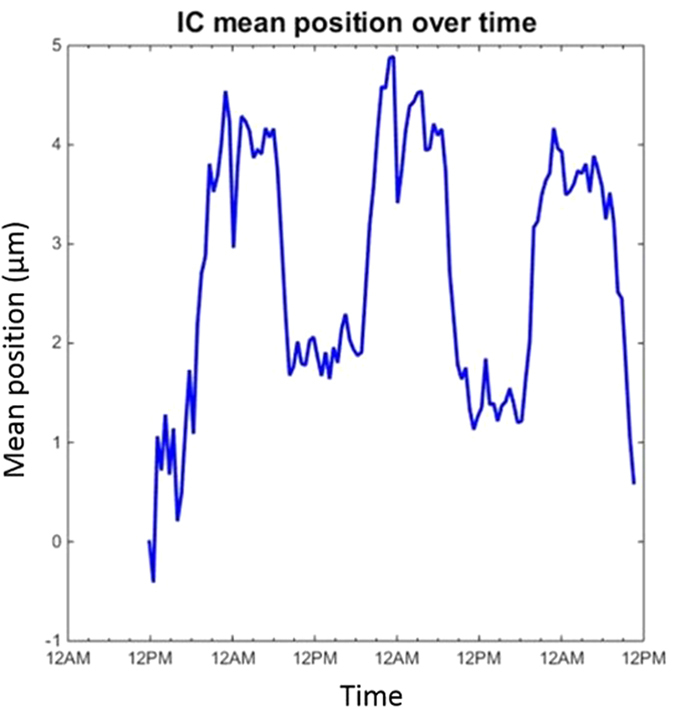
The position of the mean of the illumination curve (IC) as a function of time, taken from experimental data. The illumination curve analysis considered an area of 50 × 50 pixels in the centre of the field of view.

EI CT is suited for use with commercially available x-ray source and detector technologies, making its translation to clinical and industrial environments possible. Such environments are likely to suffer from non-ideal imaging conditions, such as temperature instabilities and vibrations of the experimental setup. As all retrieval methods described above rely on knowledge of the illumination curve, and since high-resolution CT scans can last hours, it was necessary to observe changes in the illumination curve over time. Using the experimental setup described above, we acquired and analysed multiple illumination curves (one every 15 minutes) over three days. While the shape and amplitude of the illumination curve remain reasonably constant over time, the position of the mean was found to vary in a cyclical pattern, as shown in Fig. 2. Our laboratory has no measures in place for vibration damping or temperature insulation, and the plot shown in Fig. 2 demonstrates that indeed environmental changes can have a dramatic impact on the illumination curve. To accommodate for these large drifts and ensure that images are acquired at the correct illumination positions (thus maintaining high refraction sensitivity), real-time illumination tracking was implemented into the CT scan. Here, the required spatial shift of the sample mask is found by comparing the intensity of a background region in an image to the desired value from the initial illumination curve. The positions of the sample mask and sample are then adjusted accordingly.

### Experimental Data Acquisition & Processing

A CT scan was performed using the laboratory setup described above. The source was operated at 25 mA and 40 kVp, and the exposure time per projection was 2 s. An illumination curve was acquired by recording the detected intensity as the sample mask was shifted with respect to the detector mask over one period.

The scanned sample was a rat’s heart, placed in a plastic container. The heart was harvested from an adult Sprague-Dawley rat weighing about 300 g. The rat was sacrificed by CO_2_ inhalation and cervical dislocation. Once sacrificed, a midline incision was made to completely expose the abdominal cavity and the heart was dissected free and removed. The organ was then washed with PBS and fixed in 4% PFA overnight. The heart was freeze-dried at a pressure of 10 mBar overnight in a petri dish.

The scan consisted of 360 views over 180 degrees, with 6 dithering steps per view corresponding to a resolution of approximately 13 μm in the x-direction^17^, and 3 images per dithering step. Flat-field images were acquired at each view, as they are required for the global-retrieval processing. The modified local retrieval does not require flat-field images for normalisation, and so the new procedure allows avoiding them completely in the future. The experimental data was processed using the two different phase retrieval methods (global and modified local), according to equations (5) and (6). Although the real-time illumination tracking procedure ensures that large shifts of the illumination curve are mitigated, a small translational shift of the illumination curve was observed between consecutive dithering steps. The shift was calculated for each dithering step image using equation (7), and the adjusted values were used in the modified local retrieval.

To reduce high noise levels in the absorption and scattering sinograms, a one-dimensional median filter was applied to the sinograms, using values from 8 (absorption) and 16 (scattering) neighbouring pixels. Parallel beam geometry was assumed, and CT reconstruction of absorption, scattering and phase maps was performed using FBP with the ramp (absorption, scattering) and Hilbert (phase) filters. It should be noted that for larger samples, the parallel beam approximation will not hold, and so CT reconstruction will require using a cone-beam reconstruction algorithm.

## Results

**Figure 3:**
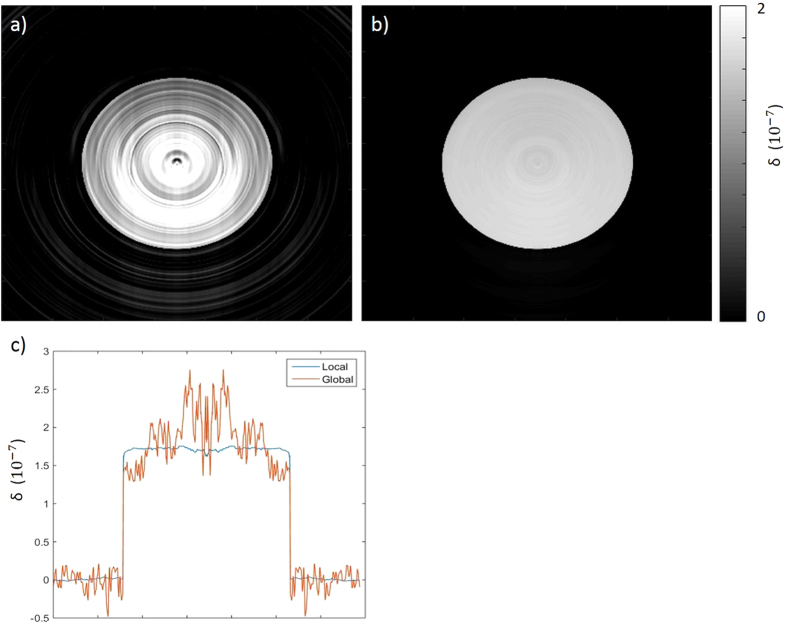
Phase maps of simulated data of a cylinder processed with global (**a**) and modified local (**b**) phase retrieval. The reduction of artefacts can be further appreciated by plotting a profile through the slices’ centre, as shown in (**c**).

Data of a cylinder, simulated with included system misalignment, was used to compare the performance of the two retrieval methods. Figure 3 presents reconstructed CT images of the sample’s phase, processed with the global (a) and modified local (b) retrieval. The plot shown (c) compares profiles taken through the centres of both slices. As expected, the reconstructed slices confirm that masks misalignment leads to significant artefacts when global retrieval is used, due to systematic errors in specific pixels. As well as ring artefacts, a gradient can be observed across the slice in Fig. 3(a). This is a result of a gradient/offset in the retrieved differential phase projections due to misalignment combined with the fact that sample rotation was over a 180 degrees range. While this could in principle be reduced by acquiring projections over 360 degrees, it is completely avoided when the modified local retrieval algorithm is used, as seen in Fig. 3(b). As well as demonstrating the removal of artefacts such as rings and gradients, the results confirm that the method is quantitative; the retrieved δ value was *δ*_*ret*_ = (1.72 ± 0.06) × 10^−7^, compared to the theoretical input value *δ*_*th*_ = 1.70 × 10^−7^. The slight variations in the locally retrieved δ profile are due to numerical errors of the fitting process used in the algorithm.

**Figure 4:**
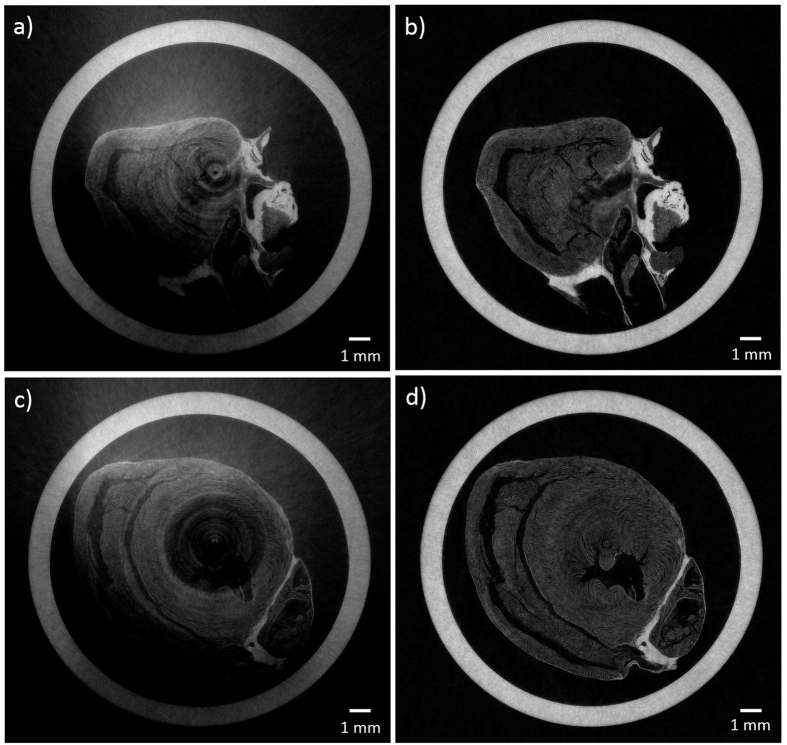
Transverse phase slices of experimental data of a rat’s heart, showing the superiority of data retrieved with the modified local method (**b,d**) compared with global retrieval (**a,c**).

Following confirmation of the effective adaptation to CT of the local retrieval approach through simulation, the method was tested on experimental data of a complex biological sample. In Fig. 4, slices reconstructed with global retrieval are shown on the left (a,c) while the figures on the right (b,d) display the same slices processed with the modified local retrieval approach. As can be seen, there is a dramatic improvement in image quality. Areas of severe masks defects can cause complete loss of information (as seen in the central region in Fig. 4(c)), which can however be fully recovered by using the modified local retrieval. As predicted by simulation, the globally retrieved slices also suffer from a gradient, which is completely eliminated in the locally-retrieved slices.

**Figure 5:**
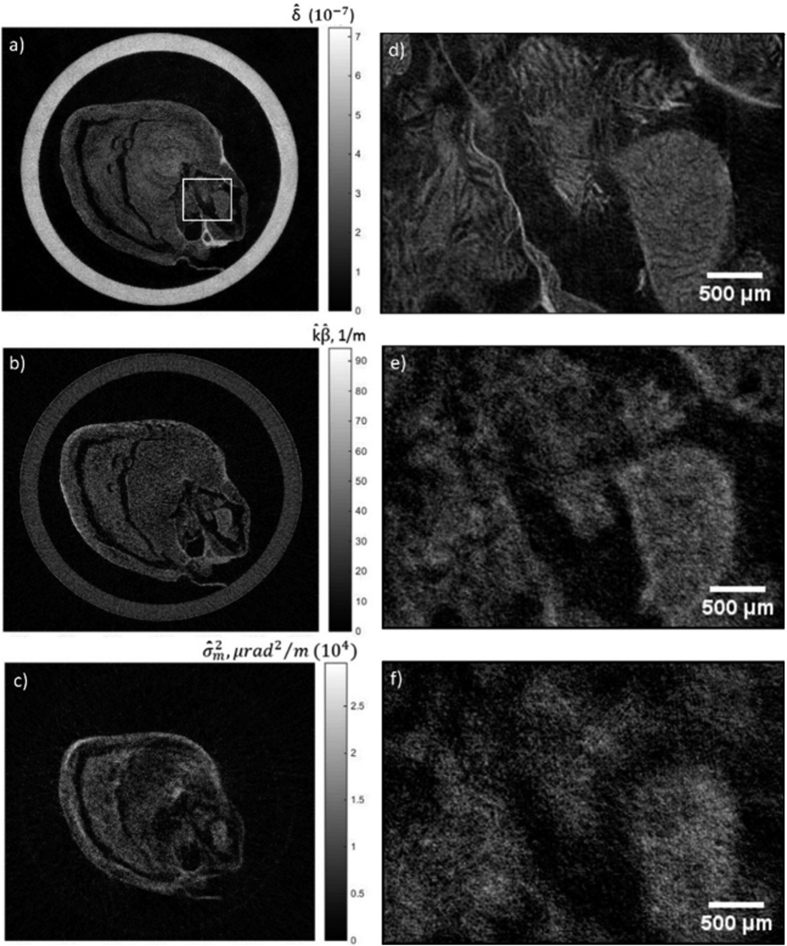
CT reconstructed slices of the phase (**a**), absorption (**b**) and scattering distribution (**c**) of a rat’s heart, processed with the modified local phase retrieval. As these data were acquired with a polychromatic spectrum, the reconstructed maps refer to sample properties estimated at effective energies, notified by the hat symbol. (**d–f**) show their corresponding magnified views of the region in the rectangle in (**a**).

It should be noted that the transition from global to local retrieval mostly affects the retrieved refraction image, while only marginally altering the absorption and scattering images. This is easily understood by considering that, when local misalignment is not taken into account, the algorithm incorrectly attributes changes in the mean position of the illumination curve to refraction by the sample. Absorption and scattering signals are determined by changes in the amplitude and width of beamlets with and without the sample, which are well-described in both cases through the use of three input frames. Figure 5 displays the results of a CT reconstruction of the phase, absorption and scattering (a–c respectively) signals of experimental data retrieved with the modified local method. Corresponding magnified views of a region in the heart (rectangle in Fig. 5(a)) are shown in Fig. 5(d–f). When comparing these three channels, it should be noted that the application of the median filter on absorption and scatter data has introduced a substantial blur to the reconstructed CT slices. However, we would like to highlight that filtering was necessary in order to improve the contrast-to-noise ratio (CNR); without filtering, most features which are now visible would have been hidden in the noise floor. For the phase slice, no median filtering was required due to its intrinsic high CNR. As a result, features in the heart are more clearly visualised in the phase slice. The scattering distribution slice provides some complementary information about regions in the sample with refractive index inhomogeneity on a scale smaller than the aperture size of the sample mask. For example, the disappearance of the cylinder in the scattering slice confirms it is a highly homogenous material. The structures seen in Fig. 5(d) appear to be the result of “cracks” in the cardiac muscle tissue, probably caused by the freeze drying process.

## Discussion

We have presented a robust phase retrieval method, capable of correcting for local misalignment and any changes to system geometry that might happen during long acquisitions. This is especially relevant for long, high-resolution CT scans, during which system parameters have been shown to vary substantially over time, as the source and/or masks can move unexpectedly, due to spontaneous vibrations and/or thermal effects. We have demonstrated via simulation that ring and gradient artefacts in phase slices are related to projections acquired with system misalignment and/or masks defects, as these create systematic errors in the retrieved quantities. The modified local retrieval method is able to correct for these systematic errors, resulting in the removal of artefacts and therefore a significant improvement in the quality and quantitativeness of reconstructed images. When applied to experimental data, locally-retrieved phase slices were free of artefacts, even in areas where the globally-retrieved slices had rings with 100% intensity variation.

While it is common practice to acquire flat-field images before and/or after a scan, until now in EI CT, flat-field images were usually taken at each rotation angle, as has been done here for the “global” processing. In situations where there are mild changes to system parameters over time, this approach helps minimising normalisation problems. However, as has been shown in this manuscript, here some parameters were heavily time dependent, and so the use of multiple flat-field images was not sufficient to improve image quality. In contrast, the new retrieval method provides improved image quality, while requiring no flat-field images, thereby reducing the EI CT scan time substantially.

The proposed retrieval method is computationally intensive when compared with the global retrieval, since the fitting process has to be repeated for each pixel in the image. However, once the shift of the illumination curve is calculated using three frames per image, the pixel-wise computation implies that the rest of the process can be parallelised. Combining this with the possibility to compute in parallel different rows in the image, different projection angles and different dithering steps, a powerful computer can significantly reduce computation time.

As mentioned before, the retrieved refraction signal in EI is proportional to the derivative of the phase shift, and so reconstruction of the phase shift (and hence δ) requires one-dimensional integration (here, effectively performed by the Hilbert filter in Fourier space). This integration is considered problematic, as any local errors in the retrieved signal are then propagated and affect the image globally, resulting in artefacts^24^. This emphasises the need for a phase retrieval method which reduces the errors in the locally-retrieved signal, such as the one presented in this paper.

While the experiment reported here served as a proof-of-principle on a small biological sample, we would expect the method to perform equally well on large samples, owing to its pixel-by-pixel computation. For samples larger than the field of view, background information can be collected by acquiring flat-field images, thus potentially making the method applicable to region-of-interest tomography, although it has not yet been attempted in EI. This would require though relinquishing one of the stated advantages of the proposed method, i.e. eliminating the need to acquire flat-field images. The resolution can be further increased by using masks with a smaller aperture size and increasing the number of dithering steps used, although at the cost of longer scan duration and computation time. Nonetheless, the presented retrieval method and associated correction for time-varying components have the potential to reliably provide CT scans with a resolution of the order of 10 μm on large samples, in harsh imaging environments affected by vibrations and/or temperature changes.

## Additional Information

**How to cite this article**: Zamir, A. *et al*. Robust phase retrieval for high resolution edge illumination x-ray phase-contrast computed tomography in non-ideal environments. *Sci. Rep.* **6**, 31197; doi: 10.1038/srep31197 (2016).
